# Engineered exosomes: a promising design platform for overcoming cancer therapy resistance

**DOI:** 10.3389/fcell.2025.1608480

**Published:** 2025-08-06

**Authors:** Peng Zhang, Kai Chen, Weifeng Liu, Xiaoying Niu, Xin Wang, Jiaqiang Wang, Weitao Yao, Xiaodong Tang, Wen Tian

**Affiliations:** ^1^ Department of Bone and Soft Tissue Cancer, The Affiliated Cancer Hospital of Zhengzhou University and Henan Cancer Hospital, Zhengzhou, China; ^2^ School of Public Health, Zhengzhou University, Zhengzhou, Henan, China; ^3^ Department of Orthopaedic Oncology Surgery, National Center for Orthopaedics, Beijing Jishuitan Hospital, Capital Medical University, Beijing, China; ^4^ Musculoskeletal Tumor Center, Peking University People’s Hospital, Beijing, China

**Keywords:** engineered exosome, immunotherapy resistance, chemotherapy resistance, targeted therapy resistance, precise treatment

## Abstract

Therapeutic resistance is a formidable barrier in cancer treatment, necessitating innovative solutions to enhance drug efficacy. Exosomes, with their unparalleled biocompatibility, low immunogenicity, and robust cargo protection, have emerged as groundbreaking nanocarriers. This review unveils the transformative potential of exosomes in overcoming drug resistance - encompassing chemotherapy, targeted therapy, and immunotherapy - in a wide spectrum of tumors. Through advanced genetic and non-genetic modifications, exosomes can dramatically enhance drug targeting and cytotoxicity, offering unprecedented precision in treatment. We explore state-of-the-art exosome engineering techniques, their revolutionary applications in clinical trials, and their promise as the next Frontier in therapeutic innovation. This comprehensive review aims to capture the cutting-edge developments and future directions of exosome-based therapies, positioning them as a cornerstone of next-generation oncology.

## 1 Introduction

Cancer therapy resistance continues to pose a significant barrier to improving patient survival, leading to suboptimal responses to chemotherapy, targeted therapy, and immunotherapy across various malignancies (Katz and Shaked, 2015; Tilsed et al., 2022; Gotwals et al., 2017; Chang et al., 2021). Traditional strategies aimed at overcoming resistance, such as combination drug regimens and dose escalation, are often compromised by tumor heterogeneity, adaptive signaling reprogramming, and microenvironment-mediated protection. As a result, there is an urgent need to design delivery systems that can effectively bypass resistance mechanisms while improving therapeutic specificity and efficacy.

Extracellular vesicles (EVs) have emerged as a remarkable class of naturally occurring nanoparticles that play a pivotal role in intercellular communication (van Niel et al., 2018; Namee and O'Driscoll, 2018). EVs are classified into: exosomes (30–150 nm), which originate from the inward budding of endosomal multivesicular bodies and are released upon fusion with the plasma membrane; microvesicles (100–1,000 nm), which are generated through outward budding of the plasma membrane; and apoptotic bodies (500–2000 nm), which are formed during the terminal stages of programmed cell death (van der Pol et al., 2012).

Among these, exosomes demonstrate the most promising characteristics for therapeutic development. In contrast to microvesicles and apoptotic bodies, which exhibit greater heterogeneity and unpredictable immunogenicity, exosomes are distinguished by their superior biostability, inherent tropism mediated by tetraspanins and integrins, and low immunogenicity, rendering them ideal carriers for precision medicine applications (Gurung et al., 2021; Zhang Y. et al., 2020). Their endosomal origin ensures a more homogeneous composition, while their nanoscale size enhances deep tumor penetration through the enhanced permeability and retention. Additionally, their membrane structure can be readily engineered for drug encapsulation, genetic editing payloads, or ligand-based targeting strategies (Zhang et al., 2015; Sadeghi et al., 2023).

While EVs as a broad category offer significant biological and translational insights, engineered exosomes emerge as a promising next-generation tool to address cancer therapy resistance. Their ultra-low immunogenicity, exceptional capacity to cross biological barriers, and significant potential for both autologous and allogeneic applications further enhance their attractiveness for clinical translation.

In this review, we firstly examined the molecular intricacies of chemotherapy resistance, focusing on how engineered exosomes can deliver drugs with enhanced precision, evade drug efflux pumps, and disrupt intracellular resistance mechanisms (Yang E. et al., 2020; Fidelle et al., 2023). Next, we explored the conundrum of targeted therapy resistance, in which engineered exosomes present a novel paradigm. By tailoring their surface ligands and modifying their cargo, these vesicles can target aberrant signaling pathways, effectively reinstating drug sensitivity in resistant tumors (Liang et al., 2021). Precision engineering not only addresses the current limitations of kinase inhibitors and monoclonal antibodies but also opens new avenues for synergistic treatments, combining molecular therapy with biological reprogramming. Engineered exosomes offer a dual approach in immunotherapy, in which resistance often manifests through immune evasion or checkpoint upregulation (Lu et al., 2024; Yue et al., 2023). They can be designed to enhance the recruitment and activation of immune cells while simultaneously delivering molecules that inhibit immunosuppressive pathways, therefore re-establishing immune surveillance. The capacity to manipulate both the immune system and tumor cells positions exosomes a pivotal tool in reinvigorating immune-based cancer treatments.

However, alongside their remarkable potential, significant challenges remain. The scalability of exosome production, the consistency of their engineering, and their long-term biocompatibility in human systems are critical hurdles (Zhu et al., 2020; Tenchov et al., 2022). This review not only addresses these technical and translational barriers but also proposes forward-thinking strategies to refine exosome engineering, optimize their clinical application, and overcome the limitations currently facing their widespread use. By synthesizing cutting-edge discoveries and proposing novel hypotheses, this review provides a comprehensive and forward-looking examination of how engineered exosomes can catalyze the next-generation of cancer therapeutics. With their unique adaptability, these vesicles hold the promise of revolutionizing cancer treatment, offering a dynamic, multi-functional platform capable of outpacing even the most resilient forms of drug resistance.

## 2 Molecular complexities of chemotherapy resistance

Chemotherapy resistance constitutes a complex, multifactorial, and dynamic process. A comprehensive understanding of the molecular mechanisms underlying this resistance is critical for the development of targeted interventions. This section outlines the key molecular contributors, such as non-coding RNAs, proteins, and lipids, with an emphasis on the major signaling pathways implicated in chemoresistance across various human cancers. Elucidating the operational mechanisms and interconnections of these systems offers vital insights for the design of effective therapeutic strategies, including the engineered exosomes.

### 2.1 The molecular landscape of chemotherapy resistance

Chemotherapy resistance arises from a complex web of molecular interactions, in which cancer cells evolve to escape the cytotoxic effects of drugs. Central to this are multiple molecular players, including miRNAs, LncRNAs, circRNAs, and proteins (Table 1) (Desterro et al., 2020; Wang et al., 2019; Zhang X. et al., 2020; Eptaminitaki et al., 2022). Previous studies have demonstrated that these molecules interact within the cell’s intricate signaling networks to enhance drug efflux, prevent drug-induced apoptosis, or repair chemotherapy-induced DNA damage (Garofalo and Croce, 2013; Xia et al., 2021; Peng et al., 2020). Overcoming this resistance requires an in-depth understanding of how each molecule contributes to the survival of cancer cells, thus paving the way for novel therapeutic strategies that exploit these vulnerabilities.

**TABLE 1:** Roles of molecules in chemotherapy resistance.

Molecules	Cancers	Mechanisms	Pathways	Drug resistance
miRNAs				
miR-30b (Muñoz-Galván et al., 2020)	Ovarian cancer	miR-30b/MYPT1	Hippo pathway	Cisplatin resistant
miR-379-5p (Shukla et al., 2025)	Ovarian cancer	miR-379-5p/RAD18/Polη	-	Cisplatin resistant
miR-9 (Bao et al., 2020)	Hepatocellular carcinoma	miR-9/EIF5A2	EMT pathway	Cisplatin resistant
miR-223 (Citron et al., 2020)	Breast Cancer	miR-223/E2F1	EGF pathway	Cisplatin resistance
miR-140 (Lu et al., 2020a)	Breast Cancer	miR-140/YY1	-	Cisplatin resistance
let-7d-5p (Wang et al., 2025)	Breast Cancer	let-7d-5p/ATG16L1	mTOR pathway	Paclitaxel resistance
miR-141-3p (Tao et al., 2025)	Breast Cancer	miR-141-3p/Rab10	Autophagy	Paclitaxel resistance
miR-30a-3p (Georgikou et al., 2020)	Pancreatic cancer	miR-30a-3p/Cx43	-	Gemcitabine resistance
miR-182-5p (Seidl et al., 2020)	Lung adenocarcinoma	miR-182-5p/GLI2	Hedgehog signaling pathway	Cisplatin resistant
miR-149-3p (Liang et al., 2020)	Colorectal cancer	p53/miR-149-3p/PDK2	Glucose metabolic pathway	Fluorouracil resistance
miR-224-5p (Yan et al., 2025a)	Colorectal cancer	miR-224-5p/S100A4	-	Fluorouracil resistance
miR-544a (Lee et al., 2025)	Oral squamous cell carcinoma	miR-544a/MTMR6	-	Cisplatin resistance
miR-424-5p (Fukumoto et al., 2024)	Bladder cancer	miR-424-5p/CCNE1	-	Cisplatin resistance
LncRNAs				
LncSNHG12 (Lu et al., 2020b)	Glioblastoma	LncSNHG12/miR-129-5p	MAPK/ERK pathway	Temozolomide resistance
Linc01956 (Liao et al., 2024)	Glioblastoma	Linc01956/MGMT/CHK1	-	Temozolomide resistance
Linc00942 (Yang et al., 2024a)	Glioblastoma	Linc00942/TPI1 and PKM2/Sox9	-	Temozolomide resistance
Lnc00461 (Qu et al., 2020)	Rectal cancer	Lnc00461/miR-593-5p/CCND1	-	Cisplatin resistance
LncSNHG5 (Lin et al., 2020; Wang et al., 2020a)	Ovarian cancer/ Acute myeloid leukemia	LncSNHG5/miR-23a LncSNHG5/miR-32/DNAJB9/	Autophagy	Paclitaxel resistance Adriamycin resistance
LncSNHG14 (Han et al., 2020a)	Colorectal cancer	SNHG14/miR-186/ATG14	-	Cisplatin resistance
LncLUCAT1 (Huan et al., 2020)	Colorectal carcinoma	LncLUCAT1/PTBP1	DNA damage	Oxaliplatin resistance
Linc01764 (Duan et al., 2024)	Colorectal cancer	Linc01764/hnRNPK	Glutamine metabolism pathway	Fluorouracil resistance
Linc00173 (Zeng et al., 2020)	Small cell lung cancer	Linc00173/miR-218/Etk/GSKIP and NDRG1	-	Cisplatin resistance
LncLYPLAL1 (Li et al., 2024a)	Small cell lung cancer	LncLYPLAL1-DT/BCL2/BECN1	-	Multi-drug resistance
DYNLRB2-AS1 (Chen et al., 2024a)	Nasopharyngeal carcinoma	DYNLRB2-AS1/DHX9	-	Gemcitabine resistance
MIR4435-2HG (Xie et al., 2025)	Pancreatic cancer	MIR4435-2HG/miR-1252-5p/STAT1	-	Gemcitabine resistance
Linc02323 (Chen et al., 2024b)	Gastric cancer	Linc02323/miR-139-3p	-	Fluorouracil resistance
LncOXAR (Lin et al., 2024a)	Hepatocellular carcinoma	LncOXAR/Ku70	-	Oxaliplatin resistance
circRNAs				
circ_001680 (Jian et al., 2020)	Colorectal cancer	circ_001680/miR-340/BMI1	-	Irinotecan resistance
circPDIA3 (Lin et al., 2024b)	Colorectal cancer	circPDIA3/miR-449a/XBP1	-	Oxaliplatin resistance
circCRIM1 (Hong et al., 2020)	Nasopharyngeal carcinoma	circCRIM1/miR-422a/FOXQ1	-	Docetaxel resistance
circCESRP1 (Huang et al., 2020)	Small cell lung cancer	circCESRP1/miR-93-5p	TGF-β pathway	Platinum resistance
circPVT1 (Zheng and Xu, 2020)	Lung adenocarcinoma	circPVT1/miR-145-5p	ABCC1 pathway	Cisplatin/Pemetrexed resistance
circ_0125,356 (Du et al., 2025)	Non-small cell lung cancer	circ_0125356/miR-582-5p/FGF9	Wnt pathway	Gemcitabine resistance
circRNF13 (Xu et al., 2025)	Oral cancer	circRNF13/IGF2BP1	m6A modification	Cisplatin resistance
circPLPP4 (Li et al., 2024b)	Ovarian cancer	circPLPP4/PIK3R1	-	Cisplatin resistance
circNUP50 (Zhu et al., 2024)	Ovarian cancer	circNUP50	p53 pathway	Cisplatin resistance
circBNC2 (Pan et al., 2025)	Prostate cancer	circBNC2/miR-4298/ACSL6	Ferroptosis	Cisplatin resistance
circFAM193 B (Yang et al., 2024b)	Acute myeloid leukemia	circFAM193B/PRMT6/ALOX15/	-	Cytosine arabinoside resistance
circ_0008315 (Fei et al., 2024)	Gastric cancer	circ_0008315/miR-3666/CPEB4	-	Cisplatin resistance
circ_0075829	Pancreatic cancer	circ_0075829/miR-326/GOT1	-	Gemcitabine resistance
Proteins				
AKT (Liu et al., 2025a)	Osteosarcoma	AKT phosphorylation/SOX2 ubiquitinoylation	-	Cisplatin resistance
CENPU (Sun et al., 2025)	Glioblastoma	CENPU/TRIM5α/RPS3 ubiquitination	-	Temozolomide resistance
CD146 (Yan et al., 2025b)	Hepatocellular carcinoma	CD146	JAG2-NOTCH pathway	Cisplatin resistance
FOSL1 (Liu et al., 2025b)	Pancreatic cancer	FOSL1/HMGA1	-	Multi-drug resistance
ZFP64 (Zhang et al., 2025a)	Breast cancer	ZFP64/GCH1 and FTH1	Ferroptosis	Doxorubicin resistance
ZC3H13 (Huang et al., 2025)	Breast cancer	ZC3H13/KCNQ1OT1/H3K4me1/2/3	-	Doxorubicin resistance
CRKL (Chen et al., 2025)	Melanoma	CRKL/PI3K/AKT and NLRP3/GSDMD	-	Cisplatin resistance
STAT3 (Dai et al., 2025)	Bladder urothelial carcinoma	STAT3/BCL-xL Axis	-	Cisplatin resistance
HDGF (Su et al., 2025)	Colorectal Cancer	HDGF/H3K36me3/CtIP	-	Fluorouracil resistance

### 2.2 Role of mRNAs in drug metabolism and transport

mRNA-mediated regulation plays a crucial role in chemotherapy resistance primarily through the expression of drug-metabolizing enzymes and efflux transporters (Fabbri et al., 2021; Lin and Kuang, 2024; Shao et al., 2015). Genes encoding multidrug resistance proteins (MRPs) (Mahaffey et al., 2009; Di Giacomo et al., 2019; Noma et al., 2008) and P-glycoprotein (P-gp) (Zhang L. et al., 2022; Shen et al., 2014) are tightly regulated by mRNAs, which, when overexpressed, lead to enhanced drug efflux, reduced intracellular drug concentration, and diminished cytotoxic effects of chemotherapy. The upregulation of mRNAs coding for DNA repair proteins, such as BRCA1 (Zhu et al., 2023; Xu Z. et al., 2020; Qin et al., 2017) and RAD51 (Short et al., 2011; Krumm et al., 2016; Liu et al., 2019), further exacerbates resistance by enabling cancer cells to repair the DNA damage inflicted by chemotherapy agents, such as cisplatin and doxorubicin. Such molecular overexpression forms the backbone of acquired resistance in various cancers, highlighting the necessity of therapies that target the transcriptional regulation of these key players.

### 2.3 The role of microRNAs as post-transcriptional regulators of chemoresistance

microRNAs (miRNAs) are small, non-coding RNAs that exert profound effects on chemotherapy resistance by post-transcriptionally modulating gene expression during cell survival, apoptosis, and drug metabolism (Rastgoo et al., 2017; Leonetti et al., 2019; Iqbal et al., 2019). For instance, miR-21 targets tumor suppressor genes such as PTEN, thereby promoting the activation of the PI3K/AKT survival pathway, which allows cancer cells to evade drug-induced apoptosis (Kim EH. et al., 2024; Fu et al., 2017; Cao et al., 2017; Chen et al., 2018). Similarly, miR-155 (Pouliot et al., 2012; Van Roosbroeck et al., 2017) and miR-221 (Xu et al., 2019; Fornari et al., 2017; Wang et al., 2016) are known to modulate key resistance pathways, including those related to the expression of apoptosis inhibitors and DNA repair enzymes. miRNAs act as critical switches in therapeutic resistance by fine-tuning the balance between pro- and anti-apoptotic signals, making them prime targets for therapeutic intervention.

### 2.4 Master regulators of long non-coding RNAs in chemoresistance

Long non-coding RNAs (lncRNAs) have gained increasing attention as master regulators of gene expression, acting through various mechanisms, such as chromatin modification, transcriptional control, and post-transcriptional processing (Singh et al., 2022; Gao et al., 2023; Han M. et al., 2020). lncRNAs, such as HOTAIR (Raju et al., 2023) and MALAT1 (Hou et al., 2023), are often overexpressed in resistant tumors, where they influence a wide array of resistance-associated processes. For instance, HOTAIR promotes epigenetic silencing of tumor suppressor genes through PRC2 recruitment, driving chemotherapy resistance by enhancing cellular survival (Yang E. et al., 2024; Ling et al., 2017). On the other hand, MALAT1 modulates alternative splicing events and promotes the epithelial-mesenchymal transition (EMT), an essential process linked to resistance and metastasis (Gupta et al., 2024; Li et al., 2017; Mao et al., 2021). The versatility of lncRNAs in modulating diverse signaling pathways highlights their central role in chemoresistance, making them attractive candidates for targeted therapies aimed at disrupting resistance networks.

### 2.5 Circular RNAs sponging miRNAs to sustain resistance

Circular RNAs (circRNAs) function as molecular sponges that sequester miRNAs and prevent them from inhibiting their target mRNAs (Xu X. et al., 2020; Cui et al., 2020; Ma et al., 2020). Overexpression of numerous circRNAs in chemoresistant cancer cells plays a crucial role in the maintenance of oncogenic pathways by inhibiting tumor-suppressive miRNAs. For instance, circHIPK3 enhances resistance by sequestering miR-637 and miR-485-3p, thus hindering its ability to downregulate anti-apoptotic proteins such as Bcl-2 (Zhang et al., 2019; Lai et al., 2020). The circRNA/miRNA/mRNA axis represents a finely tuned regulatory mechanism that cancer cells exploit to escape chemotherapy-induced cell death, offering a new layer of complexity to resistance biology.

### 2.6 Protein and lipid-mediated mechanisms of chemotherapy resistance

Proteins and lipids serve as essential components of the cellular response network that facilitates tumor cells in evading the cytotoxic effects of chemotherapy. These macromolecules contribute to drug resistance through a variety of interconnected processes, including detoxification, efflux, DNA damage repair, and inhibition of apoptosis.

Among the detoxification systems, glutathione S-transferases (GSTs) play a critical role by catalyzing the conjugation of electrophilic anticancer agents with glutathione. This enzymatic modification enhances drug hydrophilicity, thereby promoting their inactivation and elimination, which ultimately diminishes therapeutic efficacy (Lv et al., 2023; Pljesa-Ercegovac et al., 2018).

The Bcl-2 family of anti-apoptotic proteins, particularly Mcl-1 (Leber et al., 2018; Song et al., 2020), Bcl-2, and Bcl-xL (Lopez et al., 2022; Haselager et al., 2021), are pivotal regulators of the mitochondrial (intrinsic) apoptotic pathway. These proteins inhibit mitochondrial outer membrane permeabilization (MOMP) by sequestering pro-apoptotic members like Bax and Bak, thus preventing cytochrome c release and subsequent caspase activation. Their upregulation enables cancer cells to survive under genotoxic stress, rendering them resistant to apoptosis-inducing chemotherapeutics.

Membrane lipids, especially phosphatidylserine (PS) and sphingolipids, also contribute to chemoresistance by modulating plasma membrane composition, fluidity, and curvature. Changes in lipid raft structure influence the activity of membrane-bound proteins, including drug efflux transporters and death receptors. Notably, PS externalization has been linked to immune evasion and decreased drug uptake (Rajeev Krishnan et al., 2020; Chen Y. et al., 2024).

A major protein-mediated resistance mechanism involves the ATP-binding cassette transporter family, including ABCB1, MRP1/ABCC1, and BCRP/ABCG2 (Zhang W. et al., 2025; Mohammad et al., 2018). These transmembrane proteins harness ATP hydrolysis to actively extrude a wide spectrum of chemotherapeutic agents from the cell, thereby reducing intracellular drug concentrations and diminishing cytotoxic effects. Their expression is frequently modulated by transcriptional programs downstream of NF-κB (Walther et al., 2015; Li et al., 2019), which is commonly activated within the tumor microenvironment.

Concurrently, enhanced DNA damage repair mechanisms contribute to survival following chemotherapy-induced genotoxic stress. Tumor cells can upregulate critical DNA repair effectors such as ATM, ATR, and BRCA1/2, enabling efficient repair of DSBs and crosslinks induced by agents like platinum compounds and topoisomerase inhibitors (Brownlie et al., 2023). This elevated repair capacity compromises drug efficacy and fosters resistance, particularly in pancreas cancer (Crowley et al., 2021) and triple-negative breast cancer (Geenen et al., 2018). In addition to these classical repair pathways, translesion synthesis (TLS) represents an error-prone DNA damage tolerance mechanism (Shilkin et al., 2020a). This process is mediated by specialized low-fidelity DNA polymerases, which enable the continuation of DNA replication at sites of chemotherapy-induced damage, thereby preventing replication fork stalling, but at the expense of an elevated mutation rate (Patel et al., 2021; Shilkin et al., 2020b). By inducing genomic instability, TLS contributes to the survival of tumor cells, such as colorectal tumor cells (Das et al., 2024) and prostate tumor cells (Williams et al., 2024), under the selective pressure of drug treatment, ultimately promoting the development of chemotherapy resistance.

Lastly, evasion of apoptosis remains a defining characteristic of resistant tumors. Downregulation or functional inhibition of pro-apoptotic proteins such as Bax, Bak, and Caspase-3, coupled with persistent activation of survival signaling pathways - including PI3K/AKT/mTOR, MAPK/ERK, and NF-κB - collectively shift cellular fate toward survival rather than death (Li et al., 2025; Mok et al., 2022). These pathways also enhance the expression of effector proteins involved in metabolism and transcription, further consolidating the resistant phenotype.

Together, the protein- and lipid-mediated mechanisms do not operate independently but instead form an interdependent cooperative resistance network that supports tumor survival under therapeutic pressure. Consequently, therapeutic strategies aimed at reversing resistance must target this multifactorial framework, either by employing combinatorial regimens or engineered exosomes delivery platforms.

### 2.7 Epigenetic modulation in chemotherapy resistance

Epigenetic alterations - heritable changes in gene expression that occur without modifications to the DNA sequence - play a pivotal role in the development and maintenance of chemotherapy resistance across a wide range of malignancies. These epigenetic modifications, which encompass DNA methylation, histone tail modifications, and chromatin remodeling, dynamically regulate gene accessibility and transcriptional activity, thereby influencing tumor cell phenotype, survival, and drug responsiveness.

Aberrant DNA methylation is one of the most prevalent epigenetic mechanisms implicated in resistance. Hypermethylation of CpG islands within the promoter regions of tumor suppressor genes such as PTEN (Maeda et al., 2015), MLH1 (Geissler et al., 2024), and CDKN2A (Wang et al., 2024) leads to their transcriptional silencing, thereby promoting cell survival, impairing DNA damage response, and facilitating evasion of apoptosis (Ogino et al., 2007; Hiraki et al., 2010).

Histone modifications, including acetylation, methylation, phosphorylation, and ubiquitination of histone H3 and H4 tails, further modulate chromatin architecture and transcriptional activation. Histone deacetylation induces chromatin condensation and gene silencing, which can downregulate DNA repair inhibitors or pro-apoptotic mediators, supporting a resistant phenotype (Yang WB. et al., 2020; Bhaskara, 2015). Conversely, aberrant histone methylation, as H3K27me3 catalyzed by EZH2, is associated with transcriptional activation, a process linked to multidrug resistance (Marsolier et al., 2022; Yamagishi et al., 2024; Xiong et al., 2022).

Overall, the plasticity of the epigenome enables cancer cells to adapt to therapeutic stress and develop drug-tolerant states without necessitating genetic mutations. Consequently, targeting epigenetic regulators represents a compelling strategy for overcoming resistance and achieving sustained responses when combined with chemotherapy or advanced engineered exosomes delivery systems.

## 3 Unique biological features of exosomes in tumor therapy

### 3.1 Mimicking natural communication of exosome surface signatures

**FIGURE 1:**
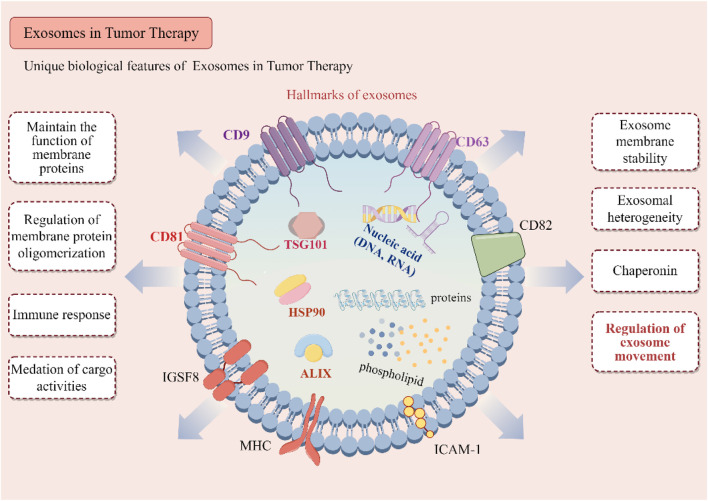
Biological characteristics of exosomes. Key surface proteins, including CD9, CD63, CD81, and CD82, are illustrated for their roles in maintaining membrane protein stability, facilitating oligomerization, and modulating immune responses. Intra-vesicular proteins such as ALIX, TSG101, HSP90, and various molecular chaperones are involved in cargo sorting and the biogenesis of exosomes. The exosomal cargo, which encompasses a diverse array of proteins, phospholipids, and nucleic acids (DNA and RNA), plays a pivotal role in mediating intercellular communication. The illustration further delineates the biological functions of exosomes, such as immune modulation, regulation of membrane proteins, mediation of cargo transfer, and their contributions to tumor microenvironment heterogeneity and exosome trafficking.

Unlike synthetic nanoparticles, exosomes are naturally derived vesicles that retain the surface signatures of their parent cells (Lai et al., 2022). This includes a wide array of surface proteins, such as tetraspanins (CD63, CD81), integrins, and major histocompatibility complex (MHC) molecules, which facilitate their interaction with target cells (Figure 1) (Salunkhe et al., 2020).

**FIGURE 2:**
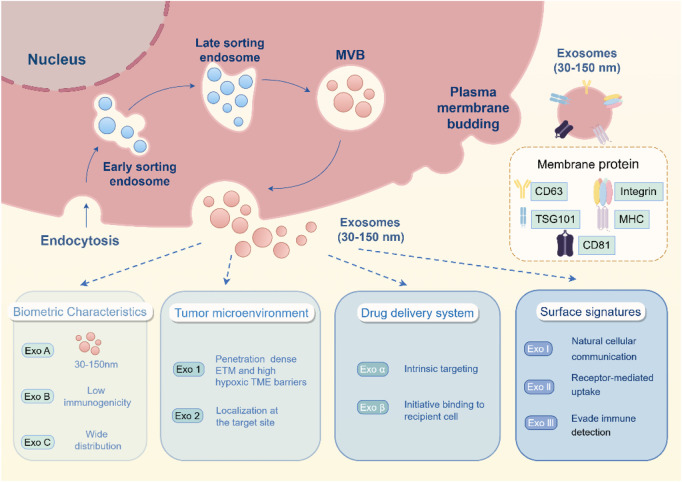
Biological properties of exosomes in tumor therapy. The endosomal pathway of exosome biogenesis begins with endocytosis, progresses through early and late endosomal stages, and culminates in the formation of multivesicular bodies (MVBs). These MVBs subsequently fuse with the plasma membrane, thereby releasing exosomes—nanoscale vesicles ranging from 30 to 150 nm in diameter—into the extracellular environment. This process is closely associated with several key features of exosomes that are pertinent to tumor biology, including their low immunogenicity, capacity to infiltrate hypoxic tumor microenvironments, and inherent targeting abilities. Additionally, the figure illustrates surface protein markers such as CD63, CD81, TSG101, integrins, and MHC, which play crucial roles in mediating intercellular communication and immune evasion. Moreover, functional domains involved in tumor microenvironment adaptation, drug delivery, and surface recognition are emphasized, underscoring the multifaceted utility of exosomes in both physiological and therapeutic contexts.

The presence of these proteins allows exosomes to engage in natural cellular communication processes, including receptor-mediated uptake and fusion with target cells (Kalluri and LeBleu, 2020; Pathania et al., 2021). This biocompatibility and ability to mimic natural cell-to-cell interactions confer exosomes with a distinct advantage in drug delivery, as they can evade immune detection and circulate for extended periods, ensuring more efficient delivery of therapeutic cargo (Figure 2) (Fu et al., 2021; Farooqi et al., 2018).

### 3.2 Tumor navigation and microenvironment penetration

The ability of exosomes to navigate complex tumor microenvironments is another critical feature that sets them apart from conventional nanoparticles. Tumors are characterized by dense extracellular matrices and hypoxic regions that create physical barriers to drug delivery (Yang M. et al., 2021). Due to their small size and flexible lipid bilayer, exosomes can penetrate these biological barriers (He et al., 2022; Li and Nabet, 2019). Furthermore, exosomes can be engineered to display peptides or proteins that enhance their ability to navigate these hostile environments, such as integrin-mediated adhesion molecules that enable them to home in on specific tumor sites (Qiao et al., 2020). In a study using exosomes to deliver small interfering RNAs (siRNAs) targeting KRAS in pancreatic cancer, researchers found that the exosomes penetrated deeply into the tumor core, significantly suppressing tumor growth (Kamerkar et al., 2017). This natural ability to traverse challenging tumor environments enhances the therapeutic potential of exosome-based delivery systems.

### 3.3 Enhancing drug delivery efficiency through natural targeting mechanisms

Exosomes also possess intrinsic targeting capabilities that can be harnessed to improve the efficiency of drug delivery (Meng et al., 2020). They contain specific receptors that facilitate the selective uptake of exosomes, enabling precise cargo delivery. For instance, exosomes derived from dendritic cells or macrophages can be engineered to express ligands that target receptors overexpressed on tumor cells, such as LFA-1 or ICAM-1, further improving the specificity of drug delivery (Guo and Jiang, 2020; Zhang W. et al., 2022). Compared with synthetic nanoparticles, which often rely on passive targeting mechanisms, exosomes can actively engage with specific cellular receptors, improving drug delivery efficiency while minimizing off-target effects (Liang et al., 2023).

## 4 Engineered exosomes for precision drug delivery and mechanism disruption

### 4.1 Engineering exosomes for targeted delivery

The specificity of engineered exosomes lies in their ability to be precisely tailored for targeted drug delivery (Tran et al., 2020; Gao et al., 2018). By functionalizing the exosome surface with ligands that bind to receptors overexpressed on cancer cells, such as EGFR, HER2, or PSMA, these vesicles can home in on tumor sites with remarkable accuracy (Jiang et al., 2024; Kim R. et al., 2024; Cheng et al., 2023). This targeted approach significantly reduces off-target effects and enhances the therapeutic index of the chemotherapeutic agents encapsulated within exosomes. Previous studies have demonstrated the efficacy of engineered exosomes engineered to carry doxorubicin directly to tumor cells, bypassing drug efflux pumps and delivering their cytotoxic payload (Liu et al., 2023; Zhu S. et al., 2022). These precision delivery systems have immense potential for overcoming the specific challenges of conventional nanoparticles.

### 4.2 Evading drug efflux pumps

One of the primary mechanisms by which cancer cells acquire multidrug resistance is through the overexpression of ATP-binding cassette (ABC) transporters, such as P-glycoprotein (P-gp/ABCB1), multidrug resistance-associated proteins (MRPs), and breast cancer resistance protein (BCRP/ABCG2) (Li et al., 2015; To et al., 2024). These transmembrane efflux pumps utilize ATP hydrolysis to actively expel chemotherapeutic agents from the intracellular milieu into the extracellular environment, thereby reducing intracellular drug concentrations below cytotoxic thresholds and compromising therapeutic efficacy. Notably, P-gp is highly expressed in various resistant tumor types and exhibits broad substrate specificity, encompassing taxanes, anthracyclines, and vinca alkaloids (Guo et al., 2024; Das et al., 2021).

Engineered exosomes possess several strategic advantages for evading recognition and export by these efflux systems (Dad et al., 2021; Chen et al., 2024d). First, the phospholipid bilayer of exosomes encapsulates their cargo - whether small molecule drugs, nucleic acids, or proteins - thereby providing a steric and structural barrier that prevents direct interaction between the therapeutic agent and membrane-bound drug efflux pumps during early cellular uptake. This cloaking mechanism ensures that the drug remains unrecognized as a substrate by efflux proteins at the initial stages of cell entry.

Second, engineered exosomes can be designed to encapsulate functional siRNAs, shRNAs, or miRNAs (Zhan et al., 2022; Shan et al., 2022) that specifically downregulate the expression of key efflux pump genes such as ABCB1, ABCC1, and ABCG2. For instance, the exosome-mediated delivery of anti-ABCB1 siRNA has been demonstrated to effectively re-sensitize drug-resistant tumor cells to standard chemotherapy regimens (Wang C. et al., 2020). This gene-silencing approach targets the fundamental cause of resistance by inhibiting the biosynthesis of efflux transporters. In resistant ovarian cancer models, exosomes engineered to deliver both paclitaxel and anti-MDR1 siRNA successfully increased drug retention and cytotoxicity, providing proof-of-concept for this dual-delivery approach (Wang C. et al., 2020).

Furthermore, exosomes exhibit intrinsic targeting capabilities through the expression of tetraspanins, integrins, and engineered surface ligands, enabling precise delivery of their cargo to specific subcellular compartments, including the perinuclear region, endoplasmic reticulum, or lysosomes (Zhu et al., 2019). These microenvironments are typically inaccessible to plasma membrane-localized drug pumps, thus providing an additional mechanism for spatial avoidance and enhancing the accumulation of cytotoxic payloads in regions where they exert maximal efficacy.

Collectively, these evasion strategies empower engineered exosomes to circumvent or functionally neutralize one of the most significant barriers to chemotherapy success: the drug efflux pump system. By integrating physical shielding, intracellular trafficking, gene silencing, and spatial targeting, exosomes serve as both stealth carriers and active modulators of drug resistance networks, offering a robust platform for restoring chemosensitivity in refractory malignancies.

### 4.3 Disrupting intracellular resistance mechanisms

The intracellular landscape of resistant cancer cells is fraught with molecular defenses that are designed to withstand chemotherapy-induced stress. Engineered exosomes can be harnessed to disrupt these defenses by delivering molecules that target the key resistance pathways. For instance, exosomes loaded with Bcl-2 inhibitors can re-sensitize cancer cells to apoptosis by negating the anti-apoptotic effects of Bcl-2 proteins (Tao et al., 2020). Similarly, exosomes can be engineered to deliver PARP inhibitors, which disrupt DNA repair pathways in cells deficient in homologous recombination, thereby inducing synthetic lethality in resistant tumors (Jung et al., 2018). Furthermore, the delivery of CRISPR/Cas9 systems via exosomes allows for precise gene editing, enabling the knockout of resistance-associated genes such as PARP1 in drug-resistant cancers (Kim et al., 2017). These strategies not only enhance the cytotoxicity of chemotherapy but also address the root molecular causes of resistance, offering a multi-pronged approach to treatment.

**FIGURE 3:**
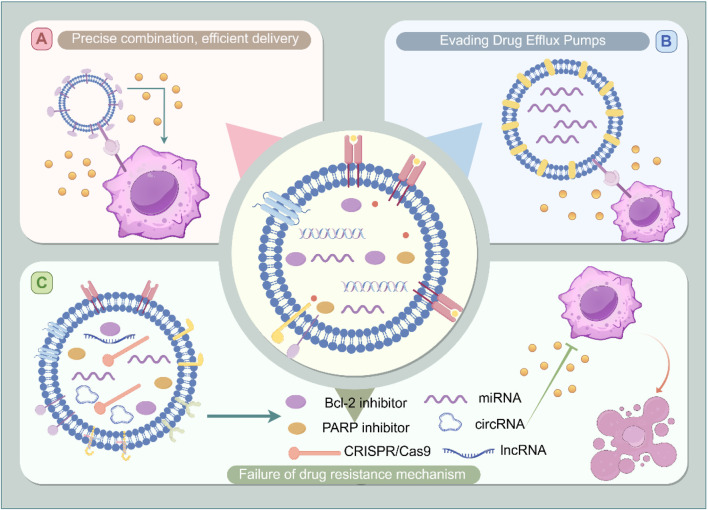
Mechanisms of engineered exosome targeting to overcome chemotherapy resistance. Subfigure **(A)** illustrates the targeted delivery of therapeutic agents via ligand-modified exosomes. Subfigure **(B)** demonstrates the capacity of exosomes to bypass drug efflux pumps, thereby enhancing intracellular drug accumulation. Subfigure **(C)** showcases exosomes loaded with a variety of therapeutic cargoes—including Bcl-2 inhibitors, PARP inhibitors, CRISPR/Cas9 systems, miRNA, circular RNA, and long non-coding RNA, which work synergistically to modulate drug resistance mechanisms. The central figure synthesizes these elements, depicting how engineered exosomes can overcome both intrinsic and acquired drug resistance by targeting key pathways and molecules associated with resistance within tumor cells.

The molecular complexities of chemotherapy resistance, driven by the intricate interplay of coding and non-coding RNAs, proteins, and lipids, present significant challenges for effective cancer treatment. Engineered exosomes offer a promising solution by providing precision drug delivery, evading drug efflux pumps, and disrupting key intracellular resistance mechanisms (Figure 3). Their unique biological features, including their natural surface signatures and ability to navigate tumor microenvironments, further enhance their therapeutic potential. As research into exosome engineering continues to advance, these vesicles hold great promise for overcoming chemotherapy resistance and improving cancer therapeutic outcomes.

## 5 Engineered exosomes-a breakthrough in overcoming targeted therapy resistance

Targeted therapies designed to inhibit specific oncogenic pathways, such as RTKs or the PI3K/AKT/mTOR axis, often encounter resistance due to downstream signaling components mutations or compensatory activation of alternative pathways. Engineered exosomes, with their inherent biological compatibility and ability to be tailored for precise therapeutic intervention, provide a novel solution for these resistance mechanisms. This section explores how exogenous surface ligand modifications and endogenous cargo alterations allow for the reinstatement of drug sensitivity in resistant tumors, opening new avenues for synergistic treatments while addressing current therapeutic bottlenecks.

### 5.1 Surface ligand modifications of exogenous exosomes for precision targeting of resistant tumors

Targeted therapies, such as monoclonal antibodies or tyrosine kinase inhibitors (TKIs), are often undermined by developing resistance mechanisms such as secondary mutations in RTKs (EGFR and HER2) or increased expression of alternative receptors such as MET and AXL (Kim et al., 2017; Fu et al., 2022). Engineered exosomes can be precisely modified on their surface to carry ligands that selectively bind to receptors overexpressed on resistant cancer cells. This ensures that the therapeutic payloads are delivered with high specificity and efficacy. For instance, anti-EGFR antibodies can be conjugated to exosome surfaces to target EGFR-mutated cancers that have developed resistance to TKIs (Toh et al., 2024). These exosomes, carrying both targeting antibodies and therapeutic cargo, can bypass the resistance mechanism by directly delivering their payload to resistant cancer cells. Similarly, HER2-targeting aptamers have been used to modify exosomes for enhanced delivery to HER2-positive breast cancer cells, overcoming resistance to HER2 inhibitors such as trastuzumab (Tao et al., 2023).

Moreover, using cell-penetrating peptides such as TAT or iRGD on exosome surfaces significantly improves their ability to penetrate deeply into solid tumors. These peptides facilitate exosome uptake by resistant cancer cells, thereby further enhancing the precision of drug delivery (Zhu Z. et al., 2022; Gan et al., 2024). For instance, iRGD-modified exosomes have demonstrated superior targeting of metastatic colon cancer, where traditional therapies have failed (Lin et al., 2021). Precision targeting through surface ligand modification not only re-sensitizes resistant tumors but also minimizes off-target effects, which are often a limiting factor in conventional targeted therapies.

### 5.2 Disrupting resistance pathways of endogenous cargo modifications

While surface modifications enable exosomes to selectively target resistant cancer cells, the actual therapeutic potential lies in their ability to carry and deliver diverse types of cargo that disrupt the underlying resistance mechanisms. By loading exosomes with siRNAs, miRNAs, or CRISPR/Cas9 gene-editing tools, cancer cells can be reprogrammed at the molecular level, restoring their sensitivity to targeted therapies. One prominent example is the use of exosomes loaded with siRNAs targeting MEK or ERK. In cancers where mutations in the MAPK pathway confer resistance to BRAF inhibitors, such as melanoma, these exosome-encapsulated siRNAs can silence MEK or ERK, thereby inhibiting compensatory survival signaling and restoring the efficacy of BRAF-targeted therapies (Vella et al., 2017). Moreover, exosomes loaded with miRNAs that downregulate the PI3K/AKT/mTOR pathway, a common escape route in numerous resistant cancers, have been exhibited to reverse resistance to PI3K inhibitors (Zhao et al., 2020; Yang C. et al., 2021; Xue et al., 2024).

Another powerful application of engineered exosomes is their ability to deliver CRISPR/Cas9 systems, which can edit genes responsible for resistance. For instance, in glioblastoma, exosomes delivering CRISPR/Cas9 tools targeting mutant KRAS G12C have demonstrated the ability to knock out the mutant allele, reinstating the tumor’s temozolomide resistance (Zhao et al., 2024). This cargo flexibility offers a significant advantage over traditional drug delivery methods, which are often constrained by poor intracellular uptake or rapid drug degradation. In contrast, exosomes can deliver their cargo directly into the cytoplasm, bypassing endosomal degradation and ensuring efficient gene silencing or protein inhibition. The precise delivery of customized cargo enables engineered exosomes to tackle molecular circuits that enable cancer cells to evade targeted therapies, offering a robust solution to multidimensional resistance mechanisms.

While exogenous engineering enables direct and multifunctional functionalization of purified exosomes, endogenous engineering provides superior biocompatibility and preserves vesicle integrity. However, the latter is often constrained by lower cargo loading efficiency and cell type dependence. Future applications could potentially leverage a hybrid strategy that integrates the strengths of both methodologies.

### 5.3 Exosome-mimicking nanoplatforms - a comparative evaluation

While natural exosomes possess distinct advantages in terms of biocompatibility, biological targeting, and functional cargo delivery, challenges such as scalability and loading consistency have driven the development of EV mimetics. These synthetic or semi-synthetic vesicles are designed to replicate the structural and functional features of natural exosomes while offering enhanced control over composition and manufacturing processes.

EV mimetics encompass platforms such as liposomes, polymeric nanovesicles, cell membrane-coated nanoparticles, and extruded nanovesicles (Du et al., 2022). While some mimetics, particularly membrane-coated particles, can preserve surface proteins for homing capability, others primarily serve as physicochemical models that lack the endogenous signaling capacity of exosomes (Gangadaran and Ahn, 2020). For instance, lipid-based mimetics like PEGylated liposomes may exhibit improved circulatory stability but often fail to fully recapitulate the complex surface architecture and cargo fidelity of native exosomes (Wei et al., 2023; Kooijmans et al., 2016).

Compared with engineered exosomes, EV mimetics present several limitations (Kim et al., 2020). First, they generally lack the complete repertoire of membrane proteins, adhesion molecules, and endogenous ligands required for natural biodistribution and cell-specific uptake. Second, mimetics do not reproduce the functional RNA and protein signatures of native exosomes, restricting their role in dynamic cell signaling and therapeutic reprogramming. Additionally, certain EV mimetic systems have demonstrated higher immunogenicity and reduced *in vivo* stability, raising concerns regarding long-term safety and clinical translation. Although their production is more standardized, this standardization comes at the expense of diminished biological function. In contrast, engineered exosomes retain the complete spectrum of native exosomal functions, such as precise intercellular communication, tissue-specific tropism, and immunologic tolerance. Their capacity to incorporate functional RNAs, signaling proteins, and membrane ligands renders them inherently advantageous as carriers for bioactive therapeutics. With advancements in both endogenous and exogenous engineering methodologies, exosomes can now be tailored for enhanced targeting specificity, cargo enrichment, and immune modulation while preserving their intrinsic biological advantages.

Consequently, although EV mimetics may serve as temporary alternatives in contexts where standardization is paramount, their applications remain largely complementary. Naturally engineered exosomes provide a more extensive and promising application scope, especially within complex biological systems requiring native bio-functionality and therapeutic adaptability. Ongoing innovations in exosome bioengineering are expected to establish them as leading candidates for next-generation targeted therapy platforms.

### 5.4 Translational challenges and opportunities of engineered exosomes

Although engineered exosomes offer substantial promise in overcoming resistance to targeted therapy, several challenges must be addressed before their full potential can be realized in clinical settings. One of the bottlenecks is their inability to address tumor heterogeneity, the presence of diverse cell populations within a tumor that may exhibit different resistance mechanisms (Labrie et al., 2022). Engineered exosomes have the potential to overcome this hurdle by delivering multiple therapeutic agents simultaneously, enabling a multi-pronged attack on resistant tumors. For example, exosomes loaded with both KRAS G12D siRNAs and TP53 mRNA, can simultaneously suppress multiple resistance pathways in cancers such as pancreatic cancer (Chiang et al., 2023). This versatility allows exosomes to address not only primary resistance mechanisms but also compensatory pathways that may arise following treatment.

Another major obstacle is the scalability of exosome production (Rehman et al., 2023). Current methods of exosome isolation, such as ultracentrifugation or size-exclusion chromatography, are labor-intensive and not easily scalable for clinical-grade production (Kim et al., 2022). Automated microfluidic systems that allow for the continuous production and isolation of exosomes are being developed as a solution to this bottleneck (Zhao et al., 2022; Zhao et al., 2019).

Additionally, variability in exosome content depending on the cell source and isolation technique raises concerns regarding consistency and standardization. Another challenge is the potential immunogenicity of repeated exosome administrations (Herrmann et al., 2021). Although exosomes occur naturally, their engineered variants may elicit immune responses over time. To mitigate this, researchers have investigated the use of immunologically inert exosomes derived from hypoimmunogenic cells or artificial lipid vesicles that mimic exosomal structures. Advances in synthetic biology have also offered the possibility of creating “immune-quiet” exosomes that can circulate in the bloodstream without triggering an immune response (Tan et al., 2024; Lin et al., 2022).

In conclusion, the combination of surface ligands and cargo modifications in engineered exosomes presents a powerful new approach to overcoming the limitations of targeted therapy. While challenges remain, ongoing innovations in exosome engineering, production scalability, and safety optimization have paved the way for their clinical translation. By employing the unique properties of exosomes, such as their precise targeting, deep tumor penetration, and cargo versatility, future cancer therapies could see a paradigm shift, with engineered exosomes at the forefront of personalized and effective cancer treatment strategies.

## 6 Revolutionizing immune surveillance of engineered exosomes in immunotherapy resistance

**FIGURE 4:**
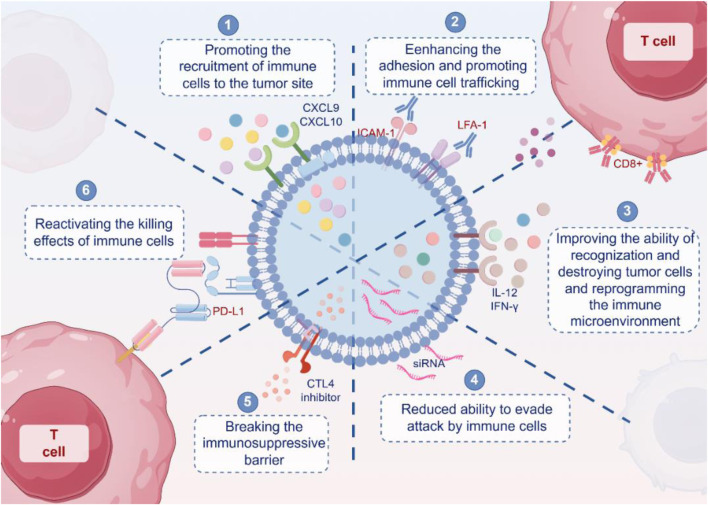
Mechanism of engineered exosomes reshaping immunotherapy. Engineered exosomes enhance anti-tumor immunity through multiple mechanisms. First, exosomes carrying chemokines such as CXCL9 and CXCL10 promote the recruitment of immune cells to tumor sites. Second, exosomes expressing adhesion molecules such as ICAM-1 and LFA-1 facilitate the migration and infiltration of immune cells into the tumor microenvironment. Third, exosomes containing cytokines such as IL-12 and IFN-γ enhance immune cell activation and recognition, while also modulating the tumor microenvironment. Fourth, engineered exosomes can reduce tumor immune escape by inhibiting immunosuppressive signaling pathways. Fifth, exosomes can serve as delivery vehicles for checkpoint inhibitors, such as CTLA-4 and PD-L1 siRNA, thereby helping to overcome immune tolerance. Sixth, immune-stimulatory exosomes can reactivate cytotoxic T lymphocytes and restore their tumor-killing capacity. Collectively, these findings illustrate how exosome engineering can reshape anti-tumor immune responses and counteract immune evasion mechanisms.

Immunotherapy offers the potential to harness the immune system of the body to combat cancer. However, numerous tumors develop resistance to immunotherapy through immune evasion mechanisms and the upregulation of immune checkpoints, such as PD-L1 or CTLA-4, which hinder the immune system’s ability to recognize and destroy tumor cells (Peng et al., 2019; Menzies et al., 2022; Pardoll, 2012). This challenge necessitates innovative solutions, and engineered exosomes have emerged as a promising platform. These nanovesicles offer a dual approach by enhancing the recruitment and activation of immune cells while simultaneously delivering molecules to inhibit immunosuppressive pathways, re-establishing immune surveillance. In this section, we investigate the potential of engineered exosomes to reinvigorate immune-based cancer treatments. We explored their role in enhancing immune cell recruitment and activation, delivering immunosuppressive pathway inhibitors, and manipulating both immune and tumor cells to overcome resistance while addressing the challenges and solutions in their clinical application (Figure 4).

### 6.1 Roles of engineered exosomes in enhancing immune cell recruitment and activation

A key aspect of immunotherapy involves enhancing the recruitment of immune cells, specifically T cells, dendritic cells, and NK cells, to the tumor microenvironment (Kubli et al., 2021; Galon and Bruni, 2019). Tumor resistance to immunotherapy often results from immune cell exclusion, in which tumors create an immunosuppressive microenvironment that impairs immune cell infiltration and activation (O'Donnell et al., 2019; Shimasaki et al., 2020). Engineered exosomes offer an innovative method for circumventing these barriers by being tailored to actively recruit and stimulate immune cells.

The design of exosomes that express surface ligands or chemokines that attract immune cells is a crucial breakthrough. Exosomes can be engineered to display ligands, such as ICAM-1 or LFA-1, which facilitate the recruitment of T cells by enhancing the adhesion and promoting immune cell trafficking to tumor sites (Nolte-'t Hoen et al., 2009; Segura et al., 2005). Additionally, exosomes can carry CXCL9 and CXCL10 chemokines, which are known for their role in attracting cytotoxic T cells, thereby amplifying the immune response against tumors (Zhang LS. et al., 2024). This targeted approach not only improves immune cell presence in the tumor microenvironment but also ensures that activated T cells reach and infiltrate the tumor mass, overcoming immune exclusion.

Another innovative approach is the delivery of immune-stimulating cytokines via exosomes. For instance, exosomes containing IL-12 or IFN-γ can facilitate the activation and proliferation of CD8^+^ cytotoxic T cells, thereby improving their capacity to identify and eliminate tumor cells (Zhang et al., 2010). These cytokine-loaded exosomes can induce a shift from an immunosuppressive to an immunostimulatory tumor microenvironment, reprogramming the immune landscape in favor of effective tumor eradication. Furthermore, exosomes carrying GM-CSF (granulocyte-macrophage colony-stimulating factor) can enhance dendritic cell activation, improving antigen presentation and boosting T-cell activation (Dai et al., 2008; Yaddanapudi et al., 2019).

This capacity of engineered exosomes to manipulate immune cell recruitment and activation forms the foundation of their role in overcoming immune resistance mechanisms. Exosomes re-establish an immune-permissive environment that supports long-term anti-tumor immunity by actively recruiting immune cells and ensuring their sustained activation.

### 6.2 Functions of engineered exosomes in rebuilding immune surveillance and inhibiting immune-suppressive pathways

While enhancing immune cell recruitment is crucial, the reactivation of immune surveillance within the tumor microenvironment depends heavily on inhibiting the immunosuppressive pathways that tumors use to escape detection. Immune checkpoints such as PD-1/PD-L1 and CTLA-4 serve as critical regulators of immune tolerance, and their upregulation in tumors leads to T-cell exhaustion and impaired immune responses (Dammeijer et al., 2020; Li Y. et al., 2024). Engineered exosomes provide a unique platform for delivering checkpoint inhibitors directly into the tumor microenvironment, thus overcoming these immune evasion mechanisms. Exosomes can be loaded with anti-PD-L1 antibodies, enabling the blockade of PD-L1 on tumor cells and the reactivation of T cell function (Poggio et al., 2019). Besides, exosomes engineered to carry CTLA-4 inhibitors can enhance the activity of regulatory T cells, shifting the balance from immune suppression to immune activation (Shi et al., 2020; Zocchi et al., 2020). Furthermore, exosomes can be designed to carry siRNAs that target immunosuppressive pathways at the gene expression level. For instance, exosomes loaded with siRNAs targeting PD-L1 mRNA can reduce PD-L1 expression on the surface of tumor cells, thereby diminishing their ability to evade T cell attack (Zhong et al., 2023).

Restoring immune surveillance through exosomal delivery of checkpoint inhibitors and gene silencing marks a significant advancement in overcoming the limitations of current immunotherapy methods. By specifically targeting the mechanisms involved in immune evasion, engineered exosomes not only increase the effectiveness of existing immune checkpoint therapies but also minimize systemic toxicity and enhance immune system activation within the tumor microenvironment.

### 6.3 Dual manipulation of immune and tumor cells

The true power of engineered exosomes lies in their ability to simultaneously manipulate both the immune system and tumor cells, creating a synergistic effect that reactivates immune-based cancer treatments. This dual approach not only enhances immune cell function but also directly influences tumor cell behavior, allowing for a comprehensive therapeutic strategy that tackles both sides of the immune evasion coin.

On the immune side, exosomes can be engineered to carry T cell-activating ligands such as OX40 L or 4-1BBL, which boost the proliferation and activation of effector T cells (Xie et al., 2013). This strategy enhances the immune response by providing additional co-stimulatory signals that overcome the exhaustion typically observed in tumor-infiltrating lymphocytes. The combination of T-cell activation and checkpoint inhibition creates a robust immune assault on the tumor, making it difficult for cancer cells to develop further resistance.

Simultaneously, engineered exosomes can deliver tumor-targeting molecules, such as TNF-related apoptosis-inducing ligand (TRAIL), which induces apoptosis in cancer cells without affecting healthy tissue (Zhang X. et al., 2024). This selective killing of tumor cells helps reduce the tumor burden while maintaining immune pressure on the remaining cancer cells. The delivery of apoptotic agents through exosomes is particularly advantageous because exosomes can evade endosomal degradation, ensuring efficient delivery to the cytoplasm of the target cells.

Despite these promising advantages, several challenges must be addressed to fully realize the potential of exosomes in immunotherapy. One major hurdle is the complexity of the tumor microenvironment, which varies widely between patients and even within different regions of the same tumor. Tumors can evolve mechanisms to resist exosome-mediated therapies, such as altering the expression of receptors required for exosome uptake. To overcome this, future exosome-based therapies may require personalized engineering, where exosomes are designed based on a patient’s specific tumor biology, ensuring that they target the most relevant immune-suppressive pathways. Scaling up the production of engineered exosomes for clinical use remains technically challenging. Although exosomes are highly biocompatible, the production process is labor-intensive and requires advanced technologies such as ultrafiltration or tangential flow filtration. Innovations in biomanufacturing, including synthetic exosomes and automated exosome production platforms, are being explored to streamline this process and make large-scale exosome therapies feasible.

In conclusion, engineered exosomes represent a groundbreaking tool for reinvigorating immune-based cancer treatment. By enhancing immune cell recruitment, inhibiting immunosuppressive pathways, and manipulating both immune and tumor cells, exosomes offer a comprehensive solution to overcome immunotherapy resistance. While challenges remain, ongoing advancements in exosome engineering, production, and personalized therapy design hold significant promise for the future of cancer immunotherapy.

## 7 Clinical applications and translational advances of exosomes

The clinical translation of extracellular vesicles, particularly exosomes, has gained significant momentum in recent years. Due to their biocompatibility, low immunogenicity, and intrinsic targeting capacity, exosomes have been extensively investigated in various therapeutic and diagnostic applications across oncology, neurology, and inflammatory diseases.

According to ClinicalTrials.gov, over 200 clinical trials have been registered to evaluate the safety, efficacy, and feasibility of exosome-based strategies (Table 2). For instance, exosomes derived from mesenchymal stem cells (MSCs) are being studied and have been tested in conditions ranging from glioblastoma (e.g., NCT03608631) to pancreatic cancer (NCT02530047). Additionally, tumor-derived exosomes are being evaluated as biomarkers for liquid biopsy and as platforms for cancer vaccines.

**TABLE 2:** Representative clinical trials of exosome-based therapies in oncology in 2024.

Clinical trial ID	Cancers	Exosome source	Application type	Phase	Status
NCT03608631	Glioblastoma	MSC-derived exosomes	Drug delivery (siRNA)	I	Completed
NCT02530047	Pancreatic Cancer	Tumor-derived exosomes	Diagnostic (liquid biopsy)	II	Active
NCT04276987	Non-Small Cell Lung CA	MSC-derived exosomes	Immunomodulation	I	Recruiting
NCT05156229	Ovarian Cancer	Engineered exosomes	Doxorubicin delivery	I	Active
NCT04127591	Colorectal Cancer	Dendritic cell exosomes	Cancer vaccine	I/II	Ongoing

In the context of cancer drug resistance, the delivery of small interfering RNAs (siRNAs) and chemotherapeutic agents via exosomes is being explored to overcome resistance pathways. For example, exosome-encapsulated formulations of paclitaxel and doxorubicin have demonstrated enhanced tumor accumulation and efficacy in preclinical models, and early-phase clinical trials are ongoing to assess their translational potential in resistant breast and ovarian cancers.

Despite these advances, several challenges remain unresolved. These include the lack of standardized GMP-grade production protocols, batch-to-batch variability, and regulatory ambiguity regarding exosome classification - as biologics, drugs, or combination products. In contrast, while EV mimetics are easier to manufacture at scale, they lack the complex surface signaling and immune interactions necessary for full biological mimicry and have yet to demonstrate comparable clinical potential.

Overall, engineered exosomes provide a unique combination of therapeutic versatility and clinical relevance that distinguishes them from other nanocarriers. The expanding body of clinical trial data highlights their potential; however, further advancements in isolation standardization, long-term safety evaluation, and regulatory harmonization will be critical for achieving widespread clinical adoption.

## 8 Conclusion

Engineered exosomes have emerged as transformative agents in combating therapy resistance in cancer, offering a multifaceted approach to enhance the effectiveness of chemotherapy, targeted therapy, and immunotherapy. By leveraging the inherent biocompatibility and versatile engineering potential of exosomes, we can precisely target and disrupt the molecular foundations of drug resistance, effectively sensitizing tumors to conventional treatments. This review has emphasized the crucial role of exosomes in delivering therapeutic agents with unparalleled specificity, modulating both tumor and immune cells, and restoring immune surveillance. Despite promising advancements, challenges such as scalable production and personalized design need to be addressed to fully exploit their clinical potential. As we continue refining exosome-based strategies and integrating cutting-edge technologies, these biological nanocarriers are poised to redefine the landscape of cancer treatment by ushering in a new era of personalized and resilient therapeutic modalities.
